# Changes in the Incidence of Congenital Anomalies in Henan Province, China, from 1997 to 2011

**DOI:** 10.1371/journal.pone.0131874

**Published:** 2015-07-10

**Authors:** Lei Xia, Lihuan Sun, Xingling Wang, Meiling Yao, Falin Xu, Guomei Cheng, Xiaoyang Wang, Changlian Zhu

**Affiliations:** 1 Department of Pediatrics, Third Affiliated Hospital of Zhengzhou University, Zhengzhou, China; 2 Department of Women and Children’s Healthcare, Third Affiliated Hospital of Zhengzhou University, Zhengzhou, China; 3 Reproduction Center, Third Affiliated Hospital of Zhengzhou University, Zhengzhou, China; 4 Perinatal Center, Third Affiliated Hospital of Zhengzhou University, Zhengzhou, China; 5 Perinatal Center, Sahlgrenska Academy, University of Gothenburg, Gothenburg, Sweden; 6 Center for Brain Repair and Rehabilitation, Sahlgrenska Academy, University of Gothenburg, Gothenburg, Sweden; Medical University of South Carolina, UNITED STATES

## Abstract

**Aim:**

To investigate changes in incidence and characteristics of congenital anomalies in infants in Henan Province of China over a period of 15 years.

**Methods:**

Population-based surveillance in Henan Province was conducted from 1997 to 2011 in 75 hospitals (40 urban districts and 35 rural counties, comprising about 20% of the total births). Basic population information was obtained from the healthcare network. All live births, intrauterine deaths after 28 weeks, and stillbirths were included. Congenital anomalies were diagnosed and reported to Henan Provincial Maternal and Pediatric Healthcare Hospital.

**Results:**

Of 1,815,920 births from 1997 to 2011, 15,660 cases of congenital anomalies were identified, resulting in an average incidence of 86.2 cases per 10,000 births. The incidence of congenital anomalies showed a significant downward trend (*p* < 0.0001) in rural areas and the whole province (*p* < 0.0001), but an increase in urban areas (*p* = 0.003). The incidence was much higher in rural than in urban areas in 1997, but this discrepancy decreased rapidly and no difference was seen between rural and urban areas in 2003. The incidence in females was higher than in males in 1997–1999 but decreased to a similar level as that in males in 2000. Maternal age exceeding 35 years was associated with a higher incidence of congenital anomalies. Among the 23 types of congenital anomalies recorded, neural tube defects were the most common; the incidence declined from 39.3 cases per 10,000 births in 1997 to 6.1 cases per 10,000 births in 2011.

**Conclusion:**

The incidence of congenital anomalies has decreased in Henan Province over the past 15 years due to significant reductions in rural areas and among girls. This decrease was partly related to a reduction in neural tube defects that was likely the result of a folic acid intervention in the province.

## Introduction

Congenital anomaly is a general term applied to structural, functional, and/or metabolic disorders that occur due to abnormal embryonic and/or fetal development. They are the main cause of infant mortality in both developed and developing countries [1,2]. The causes of congenital anomalies include genetic and environmental factors and the interactions between these factors [3–8]. More than 3,000 distinct congenital anomalies have been identified, of which 110 consist of major malformations. Twenty-three of the most common or lethal congenital malformations are monitored in China according to the requirements of the Chinese Congenital Anomalies Surveillance Program [9,10].

Congenital anomalies have significant effects on society and are a leading cause of spontaneous and induced abortion and stillbirth as well as neonatal and infant death [1,11]. Investigating changes in the incidence and leading causes of congenital anomalies is very important for evaluating the effectiveness of preventive strategies and serves as a basis for developing new preventive methods. China is a multi-ethnic and economically imbalanced country, and this has led to large variations in the incidence of congenital anomalies and neonatal and infant mortality, including pronounced differences between rural and urban areas [11]. The proportion of deaths due to congenital anomalies has increased in parallel with the reduction of deaths from asphyxia, and congenital anomalies are now among the top four causes of death in children in China [11].

Henan Province, with around 105 million inhabitants, has the largest population and is the largest agricultural province in central China. Like at the national level, there are pronounced differences between urban and rural areas in the province, as well as imbalanced economic development and a multi-ethnic population. Based on data concerning income, health expenditures, and mortality, the conditions in Henan Province are representative of China as a whole [12]. Furthermore, the average birth rate in Henan Province in 2011 was very similar to that in the whole country (11.6% and 11.9%, respectively).

The aim of this investigation was to analyze temporal and geographical trends concerning congenital anomalies and possible factors affecting these changes in Henan Province over a 15-year period from 1997 to 2011 among 1,799,062 live births, 1,224 intrauterine deaths, and 12,767 still births, as well as 2,867 terminations of pregnancy after 28 weeks due to congenital anomalies.

## Methods

A population-based congenital anomalies surveillance system was set up in Henan Province by the Chinese Ministry of Health in 1995. The system was tested in 1996 and fully implemented in 1997. A stratified sampling method was used based on the total population, birth rate, geographical location, and economic development level. The surveillance system comprises 75 sites in total, of which 40 are classified as urban and 35 as rural areas, and covers about 20% of total births in the province. This study was based on the official registration in the surveillance system and was approved by the Health Department of Henan Province. Written informed consent was not applicable in this case. The recoding information was anonymized and de-identified prior to analysis, and the analysis was approved by the Human Research Ethics Committee of Zhengzhou University.

The data presented for this report included all cases of congenital anomalies from the 75 surveillance sites in Henan Province between January 1, 1997, and December 31, 2011. These included congenital anomalies in all live births, intrauterine deaths, and stillbirths after 28 weeks of gestation as well as terminations of pregnancy after 28 weeks due to congenital anomaly. All cases were diagnosed within 7 days after the end of pregnancy. Under the guidelines of the International Classification of Disease and Related Health problems, 10^th^ Revision (ICD-10), the 23 types of congenital anomalies are included in this report. The information about each infant or fetus, including birth date, gestational age, gender, maternal age, place of residence, birth weight, anomaly diagnosis, and family history, was recorded and verified by a pediatrician or neonatologist at each hospital. Diagnoses were confirmed by physical examination and complementary examinations such as ultrasonography, X-ray, magnetic resonance imaging, chromosome analysis, or autopsy. A “congenital anomaly registration card” was filled out for each infant or fetus and reported to the Henan Provincial Maternal and Pediatric Healthcare Hospital. The card was reviewed and registered after feedback and necessary corrections. The total numbers of neonates and congenital anomaly cases at each surveillance site were calculated each year and classified according to sex, maternal age, birth/termination season, and rural or urban residence. The annual incidences for specific congenital anomalies were calculated and ranked each year.

Prenatal ultrasonography screening for structural malformation and chromosome analysis for chromosomal aberration were started in 2001, primarily in urban areas. The methods spread to the rural areas and were implemented to the same extent in both areas in 2005. Suspected severe congenital anomalies are required to be confirmed by the Provincial Prenatal Diagnostic Center. According to Chinese law, the doctor must inform the parents and suggest terminating the pregnancy in cases of diagnosed severe congenital anomalies. Pregnancies terminated at less than 28 gestational weeks due to severe congenital anomalies were not included in the current analysis.

We used SPSS 17.0 for the statistical analysis. The prevalence rate of congenital anomalies and its 95% confidence intervals (95% CI) were calculated, and chi-square tests were used for showing the differences between groups in each year. The trend in incidence was analyzed with logistic regression, and the odds ratio (OR) and its 95% CI were used for multivariate analysis. When the OR <1, the incidence of congenital anomalies was in a downward trend, and when the OR >1 the incidence of congenital anomalies was in an upward trend. The level of significance was set at < 0.05.

## Results

### Geographical location difference in the incidence of congenital anomalies

**Fig 1:**
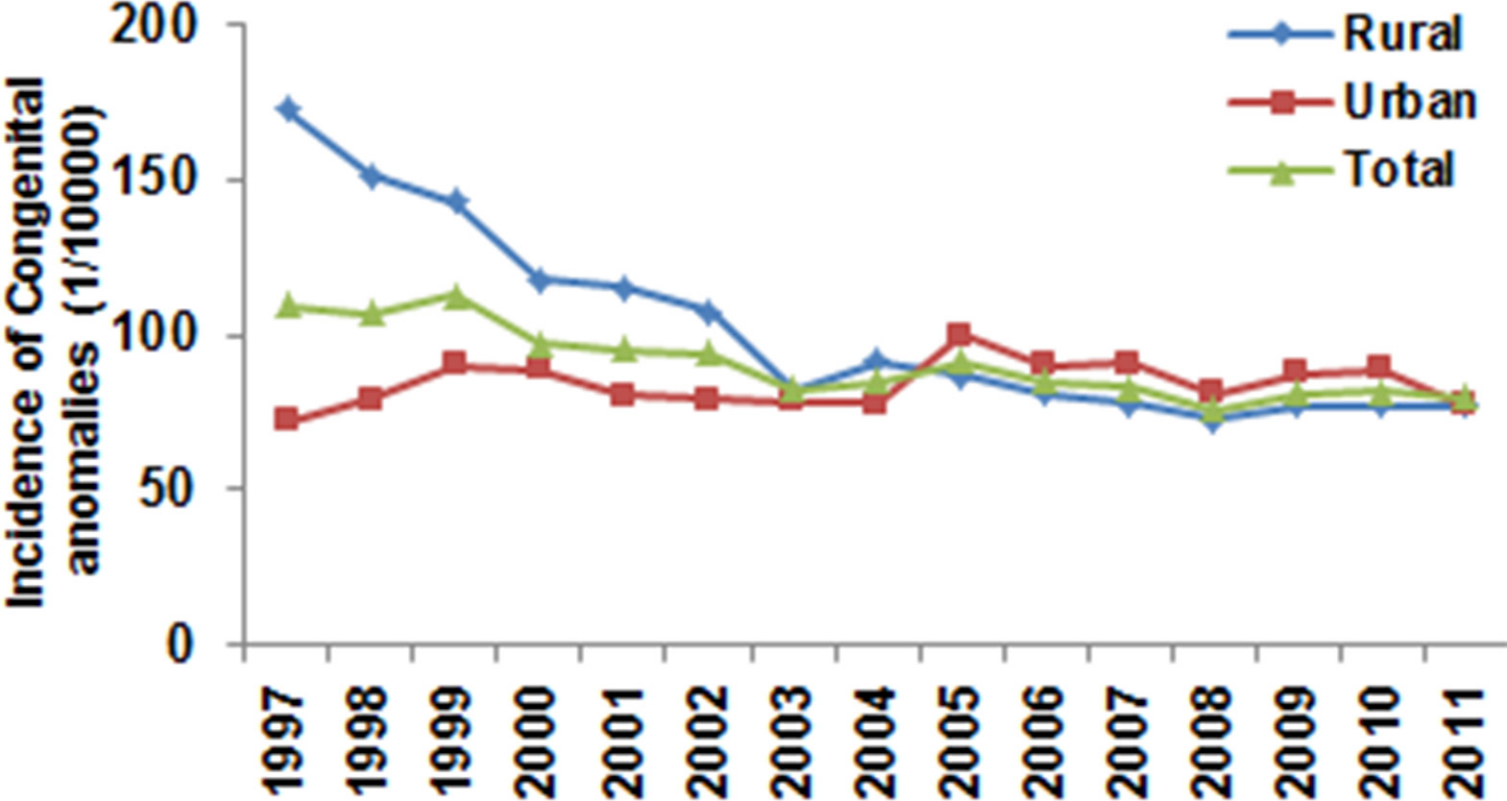
Trend in congenital anomalies in Henan Province from 1997 to 2011. The incidence of congenital anomalies in rural areas and urban areas, as well as in the whole province, was calculated each year. Logistic regression analysis showed a significant downward trend in the rural areas (*p* < 0.0001, odds ratio (OR) = 0.95; 95% confidence interval (CI) 0.94–0.95) and the whole province (*p* < 0.0001, OR = 0.98; 95% CI 0.97–0.98), but an increase in urban areas (*p* = 0.003, OR = 1.01; 95% CI 1.00–1.02). The change in incidence between 1997 and 2011 was analyzed by chi-square test in each population group, revealing that the differences in the rural population (OR = 2.26, 95% CI 1.99–2.55, *p* < 0.0001) and urban population (OR = 0.84, 95% CI 0.72–0.97, *p* = 0.021) were statistically significant.

Between 1997 and 2011, a total of 1,815,920 infants were born in the 75 surveillance sites in Henan Province, among which 15,660 congenital anomaly cases were identified, resulting in an incidence of 86.2 cases per 10,000 births. Of the 764,797 births in the urban areas, there were 6,472 cases of congenital anomalies, yielding an incidence of 84.6 cases per 10,000 births. Of the 1,051,123 births in the rural areas, there were 9,188 cases of congenital anomalies, yielding an incidence of 87.4 cases per 10,000 births. The incidence of congenital anomalies was significantly higher in the rural areas than in the urban areas (the OR = 0.97 in urban compared to rural, 95% CI 0.94–0.99, *p* = 0.045) (Fig 1). The difference was much more pronounced in 1997, when the incidence was 172.3 cases per 10,000 births in the rural areas and 72.1 cases per 10,000 births in the urban areas (the OR = 0.41 in urban compared to rural, 95% CI 0.35–0.49, *p* < 0.0001). The incidence of congenital anomalies in the rural areas decreased rapidly to 91.4 cases per 10,000 births in 2003 (the OR = 1.90 in 2003 compared to 1997, 95% CI 1.64–2.20, *p* < 0.0001) and was lower than the incidence in the urban areas in 2005. The incidence further decreased to 77.2 cases per 10,000 births in 2011 (the OR = 2.26 in 2011 compared to 1997, 95% CI 1.99–2.55, *p* < 0.0001). The logistic regression analysis showed a significant reduction in incidence in the rural areas over the studied time period (*p* < 0.0001). The incidence in the urban areas increased from 72.1 cases per 10,000 births in 1997 to 77.5 cases per 10,000 births in 2011 (the OR = 0.84 in 1997 compared to 2011, 95% CI 0.72–0.97, *p* = 0.021). The overall increase in congenital anomalies in the urban areas was statistically significant (*p* = 0.003). The total average incidence of congenital anomalies in both rural and urban areas decreased steadily, from 109.8 cases per 10,000 births in 1997 to 82.3 cases per 10,000 births in 2003 (the OR = 1.34 in 2003 compared to 1997, 95% CI 1.19–1.49, *p* < 0.001; r = −0.89, *p* = 0.007), and remained relatively stable thereafter with 80.19 cases per 10,000 births in 2011 (the OR = 1.37 in 2011 compared to 2003, 95% CI 1.25–1.51, *p* < 0.0001) (Fig 1).

The induced abortion incidence in pregnancies with severe congenital anomalies at less than 28 weeks was recorded from 2005. The incidence was 3.0 cases per 10,000 pregnancies in 2005, and this increased to 19.1 cases per 10,000 pregnancies in 2011 (the OR = 0.16 in 2005 compared to 2011, 95% CI 0.11–0.22, *p* < 0.0001). The incidence of induced abortion at less than 28 weeks increased significantly in both rural (the OR = 0.17 in 2005 compared to 2011, 95% CI 0.10–0.28, *p* < 0.0001) and urban (the OR = 0.14 in 2005 compared to 2011, 95% CI 0.09–0.22, *p* < 0.0001) areas from 2005 to 2011. Furthermore, the incidence was higher in urban than in rural areas (the OR = 2.48 in urban compared to rural, 95% CI 2.60–2.98, *p* < 0.0001). However, no fetal gender difference was observed in the rates of induced abortion at less than 28 weeks, either in urban or rural areas. The average incidence of induced abortion at more than 28 weeks was 26.9 cases per 10,000 pregnancies for males and 22.2 cases per 10,000 pregnancies for females, and 2.3% of induced abortions were of sexual ambiguity. The induced abortion was also higher in urban area (31.0 cases per 10,000 pregnancies) than in rural area (22.1 cases per 10,000 pregnancies) (the OR = 1.36 in urban compared to rural, 95% CI 1.16–1.60, *p* < 0.0001).

### Gender difference in the incidence of congenital anomalies

**Fig 2:**
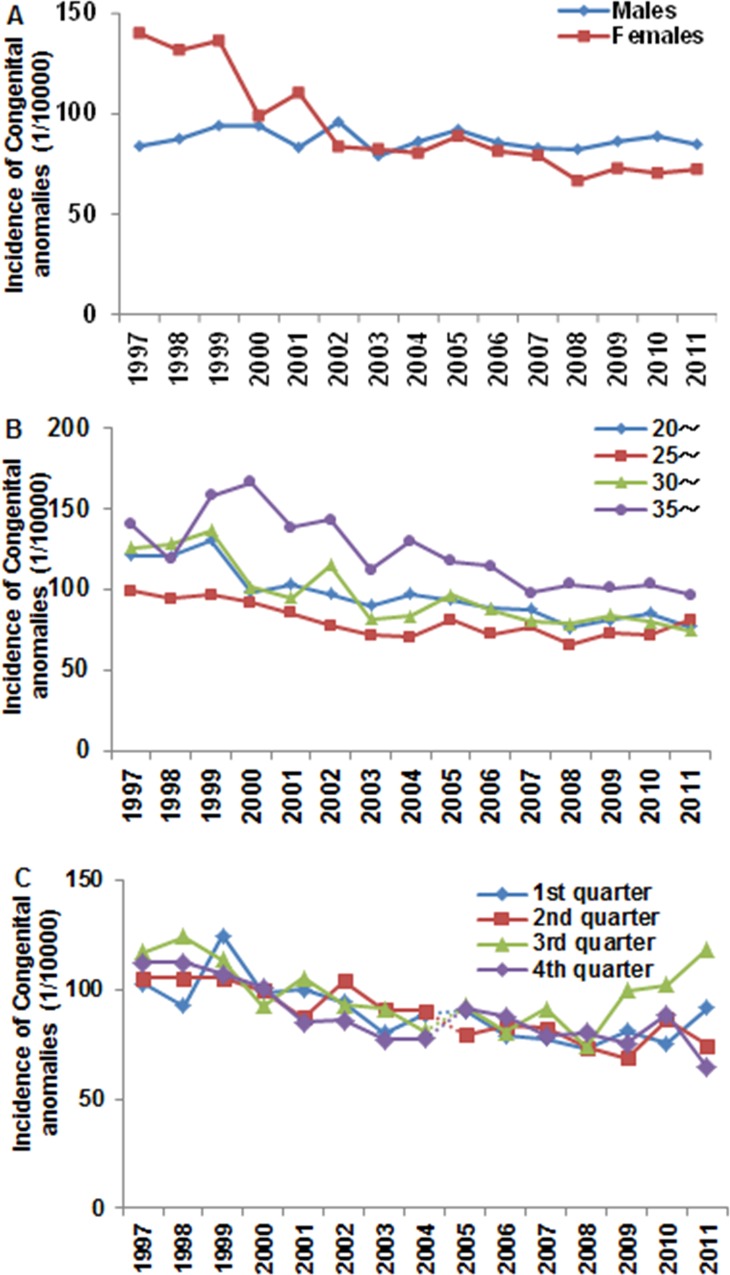
Factors associated with congenital anomalies. **A.** The incidence of congenital anomalies in males and females was calculated each year. Logistic regression analysis showed a significant downward trend in females (*p* < 0.0001, OR = 0.95, 95% CI 0.95–0.96), but not in males (*p* = 0.212, OR = 0.99, 95% CI 0.99–1.00). The difference in incidence between 1997 and 2011 was analyzed by the chi-square test in both genders, and a significant difference was observed in females (*p* < 0.0001) but not in males (*p* = 0.883). The difference in incidence between the genders in 1997 and 2011 was analyzed with the chi-square test, and a significant difference was observed in 1997 (*p* < 0.0001) and in 2011 (*p* = 0.001). **B.** The incidence of congenital anomalies at different maternal ages was calculated each year. Logistic regression analysis showed significant downward trends in all the age groups during the surveillance period (*p* < 0.0001). **C.** The incidence of congenital anomalies in the four different quarters was calculated each year. Significant reductions were seen in all seasons except the second quarter (*p* < 0.001), but no overall differences were seen between the seasons.

The incidences of congenital anomalies in boys and girls were analyzed separately (Fig 2A). A total of 1,007,012 boys and 807,963 girls were born during the surveillance period, of which 8,704 boys and 6,750 girls were diagnosed with congenital anomalies, yielding an incidence of 86.4 cases per 10,000 births in boys and 83.5 cases per 10,000 births in girls (the OR = 1.04 in boys compared to girls, 95% CI 1.00–1.07, *p* = 0.035). The incidence of congenital anomalies was 140.2 cases per 10,000 births in girls in 1997, which was higher than that in boys at 83.8 cases per 10,000 births (the OR = 0.58 in boys compared to girls, 95% CI 0.49–0.69, *p* < 0.001). The incidence in girls decreased from 140.2 cases per 10,000 births in 1997 to 83.6 cases per 10,000 births in 2002 (the OR = 1.69 in 1997 compared to 2002, 95% CI 1.43–1.98, *p* < 0.001; r = −0.87, *p* = 0.002) and to 72.3 cases per 10,000 births in 2011 (the OR = 1.95 in 1997 compared to 2011, OR = 1.95, 95% CI 1.71–2.22, *p* < 0.001; r = −0.97, *p* < 0.0001). The incidence in girls was lower than in boys from 2004 onward. The incidence in boys remained relatively stable over the study period with 83.8 cases per 10,000 births in 1997 and 84.7 cases per 10,000 births in 2011 (the OR = 0.99 in 1997 compared to 2011, 95% CI 0.86–1.14, *p* = 0.883).

### Maternal age-specific risk of congenital anomalies

The impact of maternal age on the incidence of congenital anomalies was analyzed (Fig 2B). In this surveillance population, 0.9% of the mothers were younger than 20 years, 29.6% were 20 to 24 years old, 41.4% were 25 to 29 years old, 19.2% were 30 to 34 years old, and 8.9% of the mothers were older than 35 years. The overall incidence of congenital anomalies decreased gradually, and significant differences were found between 1997 and 2011 in the 20–24 year-olds, the 25–29 year olds, and the 30–34 year olds (the OR = 1.58, 1.23, 1.62, in 2011 compared to 1997, 95% CI 1.34–1.87, *p* < 0.001, 95% CI 1.07–1.41, *p* = 0.003, and 95% CI 1.28–2.23, *p* < 0.001, respectively). The incidence was the lowest in the 25–29 year-old group (78.2 cases per 10,000 births) and highest in mothers under 20 years (122.0 cases per 10,000 births) (the OR = 0.64 in 25–29 year-old group compared to under 20 years group, 95% CI 0.55–0.73, *p* < 0.0001) followed by those 35 years or older (108.6 cases per 10,000 births) (the OR = 0.72 in 25–29 year-old group compared to under 35 years or older group, 95% CI 0.68–0.76, *p* < 0.001). The impact of delivery season on the incidence of congenital anomalies was analyzed (Fig 2C). The trend for each quarter decreased significantly between 1997 and 2011, except for the second quarter (the OR = 1.39, 1.13, 1.50, 1.46 in 2011 compared to 1997, 95% CI 1.14–1.68, *p* = 0.001; 95% CI 0.92–1.38, *p* = 0.248; 95% CI 1.24–1.82, *p* < 0.0001; and 95% CI 1.21–1.74, *p* < 0.001, respectively), but no significant differences were found when all four quarters were compared.

### Most common congenital anomalies

**Fig 3:**
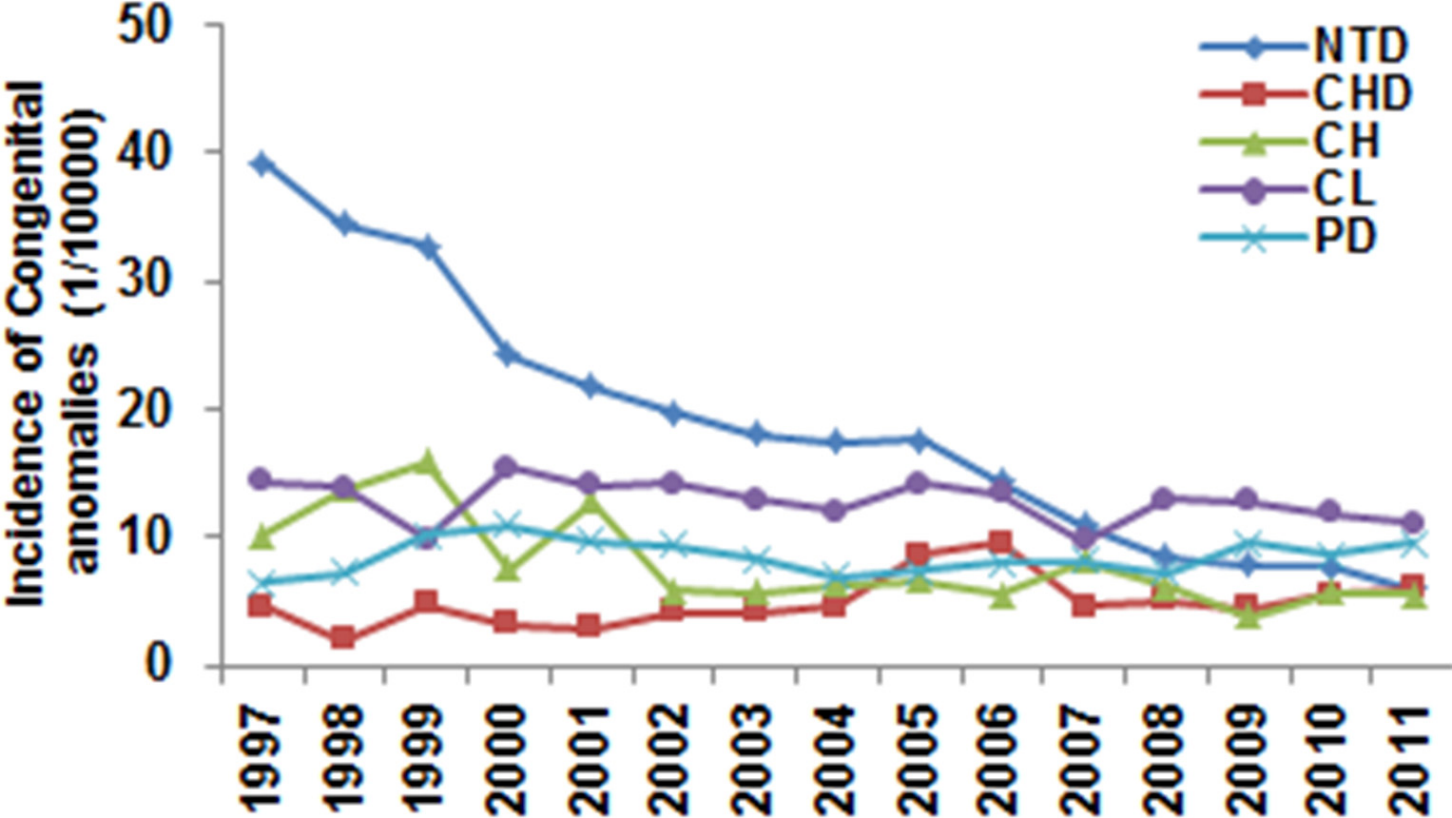
Trends in the most common congenital anomalies in Henan Province between 1997 and 2011. Incidences of the most common congenital anomalies were calculated each year. Logistic regression analysis showed a significant downward trend in NTD (*p* < 0.0001, OR = 0.88; 95% CI 0.87–0.89) and CH (*p* < 0.0001, OR = 0.94; 95% CI 0.92–0.95). There was an increase in CHD (*p* < 0.0001, OR = 1.03; 95% CI 1.01–1.05), but there was no significant change in CL (*p* = 0.141) or PD (*p* = 0.464). The difference in incidence between 1997 and 2011 was analyzed with the chi-square test, and significant reductions were observed in NTDs (*p* < 0.0001) and CH (*p* < 0.0001). NTD: neural tube defect; CH: congenital hydrocephalus; CL: cleft lip; CHD: congenital heart disease; PD: polydactyly.

The incidence of neural tube defects (NTDs, including anencephalia, myelomeningocele, and encephalocele) was 39.3 cases per 10,000 births in 1997, and this decreased to 6.1 cases per 10,000 births in 2011 (the OR = 6.48 in 2011compared to 1997, 95% CI 5.24–8.03, *p* < 0.001). The NTD incidence ranked first among all congenital anomalies between 1997 and 2007 and ranked third from 2009 (Table 1). The incidence of cleft lip and/or palate was 14.3 cases per 10,000 births in 1997 and 11.1 cases per 10,000 births in 2011, with no significant changes during the surveillance period (OR = 1.09, 95% CI 0.84–1.41, *p* = 0.536). Cleft lip ranked second up until 2008, after which it ranked first (Fig 3). The incidence of congenital hydrocephalus showed a significant decrease from 10.1 cases per 10,000 births in 1997 to 5.6 cases per 10,000 births in 2011 (the OR = 1.82 in 2011 compared to 1997, 95% CI 1.31–2.52, *p* = 0.0001). The incidence of congenital heart disease showed a significant increase (the OR = 0.53 in 1997 compared to 2011, 95% CI 0.32–0.89, *p* = 0.0001). The incidence of chromosomal aberrations (trisomies 21 and 18) decreased from 1.18 cases per 10,000 births in 2005 to 0.49 cases per 10,000 births in 2011 (the OR = 2.40 in 2011 compared to 2005, 95% CI 1.11–5.18, *p* = 0.033). The most common five birth defects were NTD, cleft lip, congenital hydrocephalus, polydactyly, and shortened limbs in 1997, while cleft lip, polydactyly, NTD, congenital heart disease, and congenital hydrocephalus were the most common in 2011 (Table 1).

**Table 1:** Rank of congenital anomalies incidence from 1997 to 2011 (cases/10,000 births).

Years	NTD			CL			PD			CH			CHD			Talipes			Limb shortening			Others		
	Rank	Rate	N	Rank	Rate	N	Rank	Rate	N	Rank	Rate	N	Rank	Rate	N	Rank	Rate	N	Rank	Rate	N	Rank	Rate	N
**1997**	1	39.3	195	2	14.33	71	4	6.46	32	3	10.13	50	7	4.64	23	4	5.2	26	5	5.04	25	—	1.0	5
**1998**	1	34.4	193	2	13.88	80	4	7.3	41	3	13.71	77	11	1.96	11	5	5.7	32	6	5.4	30	—	1.8	10
**1999**	1	32.8	197	3	9.83	59	4	10.16	61	2	15.83	95	6	4.83	29	7	4.7	28	5	5.66	34	—	3.5	21
**2000**	1	24.2	166	2	15.44	106	3	10.92	75	4	7.57	52	8	3.2	22	6	4.7	32	5	5.54	38	—	2.8	19
**2001**	1	21.83	149	2	14.12	96	4	9.67	66	3	12.89	88	9	2.93	20	6	5.4	37	5	6.3	43	—	1.5	10
**2002**	1	19.73	149	2	14.14	107	3	9.41	71	5	5.87	44	8	4.24	32	4	6.49	49	6	5.3	40	—	2.4	18
**2003**	1	17.03	154	2	12.96	111	3	8.34	71	4	5.72	49	7	4.09	35	5	4.67	40	6	4.44	38	11	1.98	17
**2004**	1	17.4	180	2	12.09	125	3	6.96	72	4	6.38	66	5	4.64	48	7	4.06	42	5	4.64	48	11	2.42	25
**2005**	1	17.66	209	2	14.2	168	4	7.44	88	5	6.59	78	3	8.7	103	7	4.06	48	10	3.89	46	9	3.80	45
**2006**	1	14.36	190	2	13.45	178	4	7.99	105	5	5.56	73	3	9.59	126	7	3.19	42	6	3.95	52	10	2.89	38
**2007**	1	10.9	172	2	9.7	153	3	8.18	129	4	8.11	128	6	4.63	73	7	4.18	66	5	4.88	77	9	3.49	55
**2008**	2	8.42	152	1	12.97	234	3	7.26	131	4	6.1	110	5	5.1	92	6	4.32	78	7	4.04	73	8	2.88	52
**2009**	3	7.82	153	1	12.83	251	2	9.56	187	8	3.83	75	5	4.45	87	6	4.19	82	7	3.99	78	4	4.7	92
**2010**	3	7.68	170	1	11.93	264	2	8.68	192	4	5.65	125	5	5.56	123	9	3.34	74	7	3.84	85	6	4.07	90
**2011**	3	6.09	148	1	11.12	319	2	9.59	233	5	5.55	135	4	6.05	147	7	3.33	81	9	3.00	73	6	4.24	103

NTD: neural tube defect; CL: cleft lip; PD: polydactyly; CH: congenital hydrocephalus; CHD: congenital heart disease; Others: other anomalies

## Discussion

In this investigation of congenital anomalies, we found a 55% decrease in the incidence in rural areas and a 19% increase in the incidence in the urban areas of Henan Province, China, from 1997 to 2011. This resulted in an overall decrease of 27% in the province. The decrease in the incidence of congenital anomalies occurred mainly in the rural areas and in girls. The most prominent decrease was reported in NTDs; indeed the decreased incidence was due almost entirely to the 84% decline in the incidence of NTDs during the surveillance period. However, prenatal diagnosis of congenital anomalies from prenatal ultrasound has seen significant progress in recent years, including 3-D ultrasound and ultrafast magnetic resonance imaging, and this has increased the sensitivity of prenatal diagnosis of major or minor structural anomalies. These prenatal diagnostic techniques are now also widely used in both urban and rural areas in China. The termination of pregnancies with severe congenital structural anomalies after prenatal diagnosis will definitely have a significant influence on the incidence of congenital anomalies. The incidence did not show dramatic change after 2005 even though the rate of induced abortion increased significantly. These results indicate that either the incidence of congenital anomalies increased or that aborted pregnancies constituted only a small portion of total congenital anomalies.

Previous studies have shown geographic and urban-rural disparities in the incidence of congenital anomalies [13]. In this study, the incidence showed a downward trend in rural areas and a smaller upward trend in urban areas. The differences in these trends might be related to diagnostic capabilities and differences in congenital anomaly types between urban and rural areas. The use of ultrasonography is increasing in rural areas during the third trimester of pregnancy, and the detection of fetal structural defects before delivery has increased, leading to more pregnancies being terminated and thus to a lower incidence of liveborn infants with congenital anomalies [14]. Improvements in maternal health care and the use of multivitamins and folic acid during pregnancy in rural areas might have contributed to the decrease in congenital anomalies [7,15]. At the same time, pollution and other modern lifestyle-related risk factors (e.g. delayed childbearing) might be contributing factors to the increase in congenital anomalies in urban areas [3,4].

When it comes to gender-related differences in cleft lip and polydactyly, these conditions have been reported to be more prominent in males, whereas NTDs and cleft palates are more common in females [16,17]. A recent population-based study in the United Kingdom found that the overall risk of congenital anomalies is greater in males than in females, and it seems that socio-demographic and maternal factors do not affect gender differences [18]. In this investigation, the incidence of congenital anomalies in females was found to be 40.2% higher than in males in 1997 (*p* < 0.0001), but no gender difference was observed in 2000, at which time the overall incidence of NTDs had decreased by 38.4% compared to 1997. The earlier gender-related difference was probably related to the high incidence of NTD, which was more prominent in girls, in 1997 [16]. Interactions between sex hormones and organ development might be possible causes of the gender differences found for some congenital anomalies [19,20].

The relationship between maternal age and congenital anomalies has been well established [21,22], and younger (less than 20 years) and advanced (over 35 years) maternal ages are related to higher incidences of congenital anomalies [6,22,23]. In our investigation, the average ratio of maternal age less than 20 years was 0.9%; this ratio showed an increasing trend and reached the highest point (1.7%) in 2011. The 25–29-year-old age group showed the lowest incidence, indicating that this is the most favorable reproductive age. At older ages, the risk of chromosomal abnormalities increases and oocytes are increasingly affected by environmental pollution and accumulated toxins [23,24], both of which are potential high-risk factors for congenital anomalies [21,22,25].

NTDs are among the most common congenital anomalies, with an incidence of about 1% (0.2–10%) of live births worldwide [26], and are considered to be a result of interactions between environmental and genetic factors [26]. In China, NTDs are the most common congenital anomalies and a major cause of stillbirth and infant mortality, accounting for approximately one in three stillbirths and one in three to one in four neonatal deaths [27]. During the surveillance period, the incidence of NTDs was highest at 3.9% in 1997, exceeding the average incidence in other provinces of China (1.6%) [28] as well as that in Korea (0.5%) [29,30]. The NTD incidence showed a downward trend from being the most common congenital anomaly in 1997 to the third most common in 2011. Periconceptional folic acid supplementation to prevent NTDs was implemented in 1993 in China, and it was found that periconceptional intake of 400 µg folic acid daily could reduce the risk of NTDs by 79% in the areas with high incidence and by 41% in the areas with low incidence [31]. However, the overall incidence of NTDs was still quite high, thus the Chinese government launched a nationwide program to increase the use of folic acid in the preconception period among the women in rural areas in 2009 [32]. Folic acid supplementation in Henan Province followed the national program of taking 400 µg folic acid daily from the time of three months prior to pregnancy to three months after pregnancy. In this study, the decline of NTDs beginning in 1997 and further decreasing in 2007 occurred during the same period as the program that actively promoted the use of low-dose folic acid supplements for pregnant women in the province. Many reports have confirmed that deficiencies in folic acid, vitamin A, vitamin B12, and zinc during the first trimester of pregnancy can contribute to NTDs, and it has been established that increased folic acid intake during pregnancy can significantly decrease NTD-related pregnancy outcomes [33–36]. The incidence of chromosomal aberrations exhibited a decreasing trend, which was probably related to the introduction of advanced noninvasive prenatal diagnostic methodology with subsequent induced abortion [37].

This report includes a long period of dynamic surveillance of congenital anomalies in the most populated province in China. The main investigators worked on this surveillance project for more than 20 years. However, there are some limitations of this study. First, this surveillance of congenital anomalies was only looking at 23 out of a total of 110 of the most frequent and the lethal anomalies. Therefore, some of the congenital anomalies might not be detected within a few days after birth, which could lead to the incidence of anomalies in this investigation being lower than the true incidence. Second, we did not have information regarding termination at less than 28 week gestation age with severe congenital anomalies before 2005, which could influence the calculation of the incidence of anomalies. Third, because this is a retrospective analysis of surveillance data starting from 1997 and there was no individual database for each pregnancy, we cannot analyze confounding factors such as multiple births, parity, smoking, drinking alcohol, socioeconomic status, medical history, prenatal care, or paternal details, nor can we analyze periodicity and time trends by using a newly developed methodological approach [38].

In summary, the incidence of congenital anomalies in Henan province has decreased dramatically in the rural areas and in females over the past 15 years, and cleft lip has replaced NTD as the most common congenital anomaly during the same period. These changes were probably related to a folic acid intervention among pregnant women in the rural areas. Maternal age is an important risk factor for congenital anomaly, and prenatal screening by ultrasonography for detecting structural malformations and gene analysis for detecting severe genetic diseases might be a second step to detect severe congenital anomalies in addition to preventive interventions among pregnant women.
